# Total thyroidectomy versus hemithyroidectomy with intraoperative radiofrequency ablation for unilateral thyroid cancer with contralateral nodules: A propensity score matching study

**DOI:** 10.1186/s40463-022-00578-6

**Published:** 2022-06-11

**Authors:** Qianqian Yuan, Lewei Zheng, Jinxuan Hou, Rui Zhou, Gaoran Xu, Chengxin Li, Gaosong Wu

**Affiliations:** Department of Thyroid and Breast Surgery, Zhongnan Hospital of Wuhan University, 169 Donghu Road, Wuhan, 430071 Hubei People’s Republic of China

**Keywords:** Papillary thyroid cancer, Benign nodules, Ablation, Surgery

## Abstract

**Background:**

For unilateral papillary thyroid carcinoma (PTC) patients with contralateral benign nodules, optimal treatment decisions are made according to patient preference and the disease’s pathological features. This study was performed to evaluate the efficacy and complications of hemithyroidectomy with intraoperative radiofrequency ablation (RFA) compared with total thyroidectomy.

**Methods:**

Patients with unilateral PTC and cytologically benign contralateral nodules were enrolled from 2014 to 2018. Total thyroidectomy or hemithyroidectomy with intraoperative RFA of the contralateral nodule was offered to patients who had anxiety regarding their disease. The operation-related parameters, transient or permanent nerve injury, hypocalcemia and disease recurrence, were recorded and compared between the two groups.

**Results:**

After propensity score matching, 191 patients who underwent total thyroidectomy and 224 contralateral nodules in 191 patients underwent hemithyroidectomy with intraoperative RFA (HTRFA) were included. The volume reduction ratios of the contralateral nodules were 67.7% at 12 months and 95.8% at 24 months. The total thyroidectomy group reported significantly higher hypocalcemia than HTRFA within one year (7.8% vs. 2.6%, *p* = 0.022). Supplemental levothyroxine was not required in 28.3% (54/191) of the patients one year after HTRFA. With a median follow-up of 4.1 years, three recurrences (1.6%) were observed in the HTRFA, and no recurrence occurred in the total thyroidectomy group (*p* = 0.246).

**Conclusions:**

Hemithyroidectomy for unilateral PTC and intraoperative RFA for contralateral nodules were acceptable and effective treatment approaches and did not increase the risk of complications.

**Graphical Abstract:**

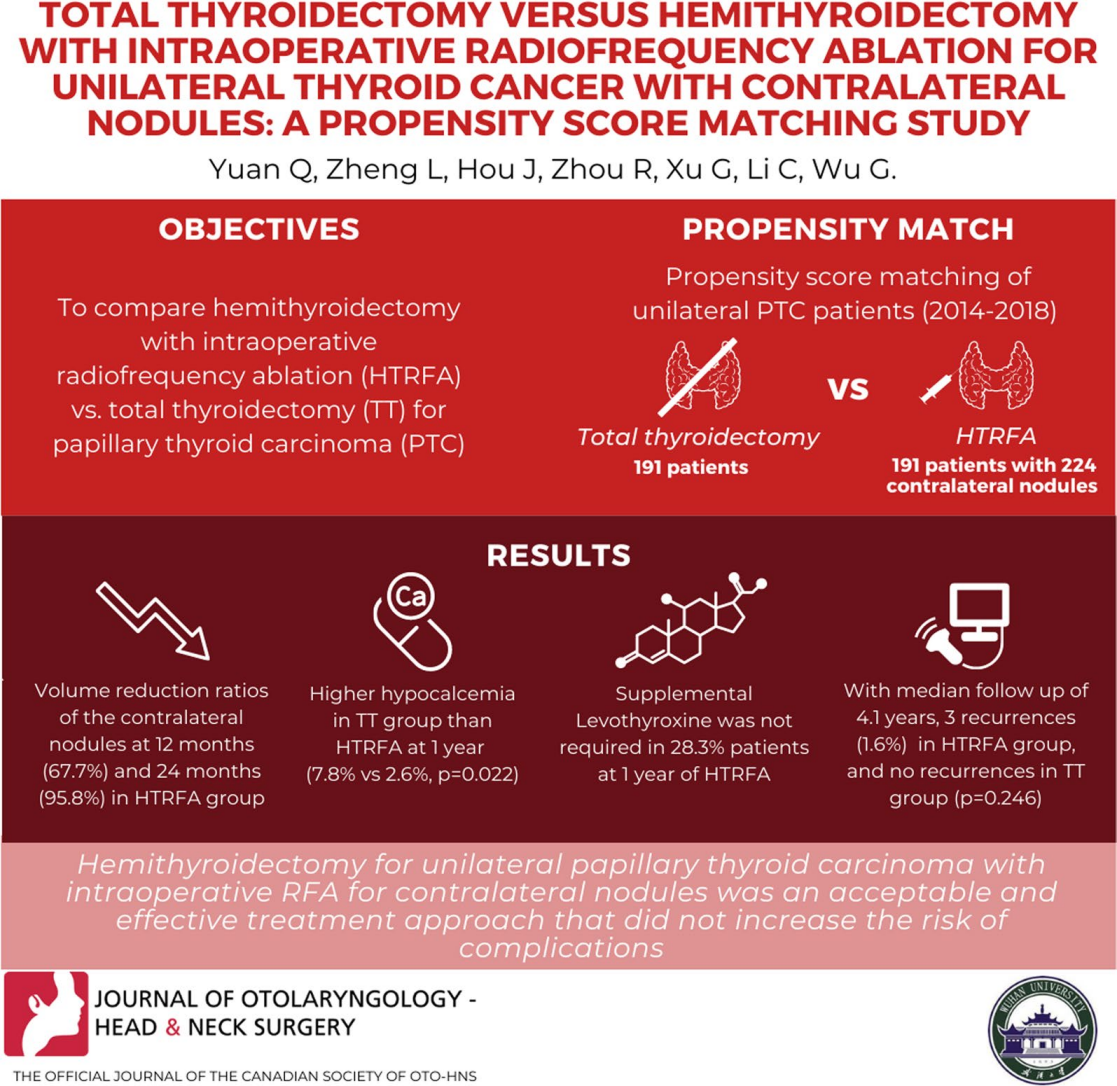

## Background

The prevalence of thyroid nodules detected by high-resolution ultrasonography can be up to 75% in randomly selected individuals [1]. Thyroid cancer occurs in 5% to 15% of patients with thyroid nodules, depending on factors such as sex, age, family history, and radiation exposure [2]. Given the increasing incidence of thyroid cancer and the high prevalence of thyroid nodules, detection of contralateral nodules by ultrasound is common in patients with cytologically proven thyroid cancer scheduled for surgery.

The treatment strategy for patients with low-risk papillary thyroid carcinoma (PTC) who have cytologically benign contralateral nodules calls into question [3]. According to the 2015 American Thyroid Association (ATA) guidelines, contralateral thyroid nodules may be a criterion for a bilateral procedure because of plans for radioiodine (RAI) therapy or to facilitate follow-up strategies or address suspicions of bilateral disease [4]. Ritter et al. investigated the natural history of nonsuspicious contralateral nodules after hemithyroidectomy for unilateral PTC, and the results revealed that hemithyroidectomy for low-risk patients with a small PTC and nonsuspicious contralateral nodules was a safe initial treatment option and was associated with a decreased occurrence of transient hypoparathyroidism [3]. However, hemithyroidectomy for unilateral PTC with contralateral benign nodules was sufficient. Patients in China tend to feel anxious about disease progression and choose to undergo total thyroidectomy to prevent occult PTC in the contralateral lobe [5].

Thermal ablation has been used to ablate benign nodules and yields satisfactory results [6–8]. Radiofrequency ablation (RFA) has been proven effective in achieving nodule shrinkage [7, 9–11]. In our institution, intraoperative RFA is an alternative method to treat contralateral benign nodules after hemithyroidectomy for unilateral PTC. This retrospective propensity score matching study based on a prospectively maintained database was designed to evaluate the feasibility of intraoperative RFA in curing disease and relieving anxiety for patients who were worried about disease progression. Total thyroidectomy and hemithyroidectomy with intraoperative RFA were compared in terms of effectiveness, oncological outcomes, and complications.

## Methods

### Patients

The study was approved by the ethics committee of Zhongnan Hospital of Wuhan University. Informed consent for treatment procedures was obtained from each patient. Unilateral PTC patients from October 2014 to October 2018 were reviewed at two tertiary referral academic medical centers, viz. Tongji Hospital of Huazhong University of Science and Technology and Zhongnan Hospital of Wuhan University. Patients who were anxious about their disease were asked to complete the hospital anxiety and depression scale (HADS) questionnaire, which was used to assess their degree of anxiety. The HADS consists of 14 items pertaining to anxiety and depression [12]. Scores for the anxiety and depression subscale ranged from 0 to 21, and anxiety subscale values exceeding seven were considered to indicate anxiety in the patient. According to the patients’ disease characteristics and preferences, total thyroidectomy or hemithyroidectomy with RFA was recommended.

Patients undergoing total thyroidectomy or hemithyroidectomy with RFA who met the following criteria were enrolled: i) Nodules were examined through preoperative ultrasound-guided fine-needle aspiration cytology (FNAC) and *BRAF* mutation analysis. If the first FNAC was indeterminate, the second FNAC would be performed. Only the contralateral nodules confirmed benign by the second FNAC were included; ii) patients with cytologically unilateral cancer and containing pathologically benign contralateral nodules; iii) no evidence of lymph node metastases (cN0) at palpation and neck ultrasound (US); iv) contralateral nodules had all three orthogonal dimensions ≤ 20 mm and located in the thyroid gland; v) the index of the contralateral benign nodules had to be solid or predominantly solid (< 30% cystic) on US [12]. vi) No more than four nodules on the contralateral lobe were treated with FNAC and RFA.

All patients were evaluated with laboratory tests (complete blood count, thyroid function tests), imaging studies including chest radiography, and US of the thyroid and cervical lymph nodes before treatment. According to ATA recommendations, the thyroid stimulating hormone (TSH) level was maintained between 0.5–2 mU/L in both groups.

### Thyroidectomy

All operations were performed by the same senior surgeons (W.G. and H.J.) with a standard technique of fine capsular en bloc dissection and resection from the inferior pole to the superior pole [13]. Intraoperative neuromonitoring was adopted in the protocol [14]. Superior parathyroid glands were identified and preserved in situ [15]. Inferior parathyroid glands were protected in situ or autotransplanted in the sternocleidomastoid muscle according to three certain types based on their blood supply and location [16]. All patients who underwent hemithyroidectomy with RFA received ipsilateral prophylactic central compartment neck dissection, and patients who underwent total thyroidectomy received ipsilateral prophylactic central compartment neck dissection on the side with the malignant lobe [17, 18]. The ipsilateral CCND entailed the removal of the prelaryngeal, pretracheal, and right or left paratracheal nodal basins.

### Hemithyroidectomy with intraoperative RFA

After thyroid hemithyroidectomy, the contralateral nodules were treated with intraoperative RFA. A bipolar RFA generator (CelonLab POWER, Olympus Surgical Technologies Europe, Hamburg, Germany) and a 9-gauge/15-gauge bipolar RF applicator with a 9/15-mm active tip were employed in our study. US-guided FNAC and RFA were performed using a Versana Premier Pt ultrasound machine, a 12 L probe for FNAC and an L8-18i probe for intraoperative RFA. The RFA power was 5 watts. During the application of RF energy, the generator continuously measures the electric impedance of the tissue between the two electrodes at the tip of the RF applicator. The power output was automatically adjusted based on the change in tissue impedance.

Intraoperative RFA was performed under general anesthesia after the completion of hemithyroidectomy. RFA was conducted over the thyroid gland. It was able to eliminate the complications that might suffer from percutaneous RFA. The “hydrodissection technique’’ was used during the ablation process to prevent recurrent laryngeal nerve injury and destruction of the trachea, carotid artery, internal jugular vein, and esophagus by heat energy. If the distance between the tumor and other critical cervical structures was < 5 mm, normal saline with 0.0005% adrenaline was first injected using a 23-gauge needle to form at least a 1-cm distance between the tumor and the critical structures to reduce the risk of thermal injury [19].

### Complications

Transient or persistent hypoparathyroidism confirmed by serum calcium levels was less than the lower limit at the examination center. Postoperative vocal cord paralysis (VCP) was defined as fixed vocal cord mobility with laryngoscopic examination. Laryngoscopic examination was performed before the operation and on the second day after surgery. If VCP was observed, postoperative laryngoscopic examination was performed every three months for the first year and then annually after that. US surveillance was performed for all the patients every three months for the first year and then annually. Postthyroidectomy hypoparathyroidism or VCP lasting more than six months was considered permanent hypoparathyroidism or VCP [20]. Locoregional recurrence was defined as cervical/superior mediastinal disease detected by US and identified by FNAC.

### Statistical analysis

We conducted propensity score matching analysis using a logit model to minimize the effects of potential confounders on selection bias. The model was adjusted for the following variables: sex, age at the time of surgery, number, size and location of primary tumors and contralateral nodules, and follow-up time. Before matching, the mean propensity score was 0.654 for patients in the hemithyroidectomy with the RFA group (*n* = 271) and 0.705 for patients in the total thyroidectomy group (*n* = 368), with a standardized difference of 19.4% (*p* < 0.001). A total of 382 patients (191 one-to-one matched patients from each cohort) were compared using the nearest neighbor method. After matching, the mean propensity score was 0.524 for patients in the hemithyroidectomy with the RFA group (*n* = 191) and 0.586 for patients in the total thyroidectomy group (*n* = 191), with a standardized difference of 4.9% (*P* = 0.174).

Recurrence-free survival and complication rates were subsequently recalculated and compared. The volume reduction ratio (VRR) was calculated as follows: VRR (%) = ([initial volume—final volume] × 100)/initial volume. Continuous variables between the groups were compared using nonparametric tests. The positive rates of the two groups were compared by the chi-square or Fisher’s exact test, if appropriate. Two-sided *p* values < 0.05 were considered statistically significant. Statistical analyses were conducted using SPSS software (version 25.0; SPSS, Chicago, IL, USA). The significance level was defined as a p value of less than 0.05.

## Results

### Patients

**Fig. 1:**
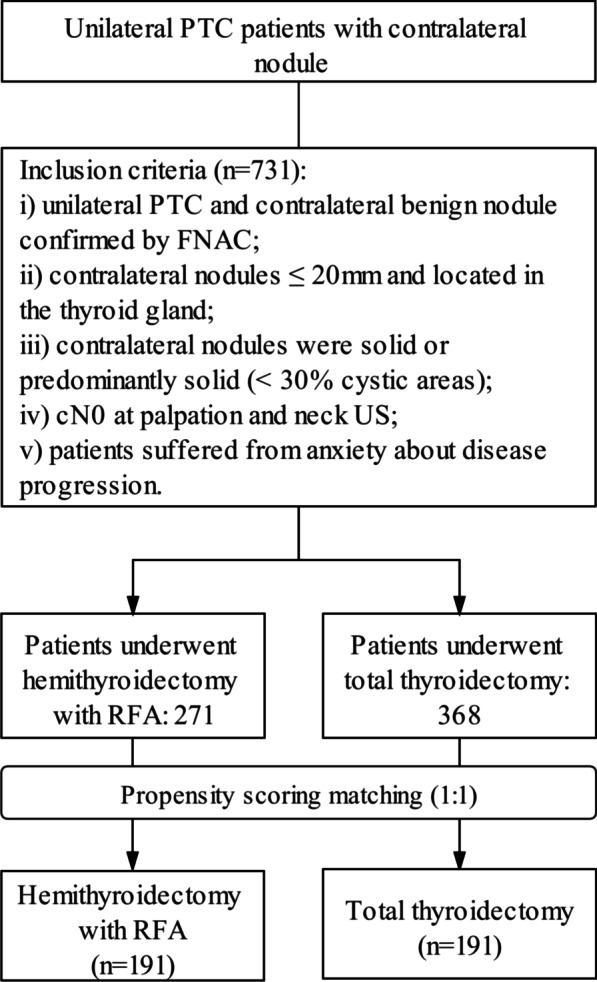
Workflow chart of the study

We reviewed a total of 731 medical records of unilateral PTC patients with contralateral nodules who underwent total thyroidectomy or hemithyroidectomy with intraoperative RFA. Fifty-one (7.0%) patients with extrathyroidal extension (ETE) and 24 (3.2%) patients with gross cervical lymph node metastases during the surgery were excluded from the study. A total of 224 contralateral nodules in 191 patients were included in the hemithyroidectomy with the RFA group after propensity score matching. Patient enrollment is presented in Fig. 1.

Baseline characteristics showed no difference between the two groups after matching in terms of mean age, sex and follow-up time (*p* = 0.364, 0.608, 0.172). The median size and volume of contralateral nodules in the two groups had no significant difference, nor did the side of the tumors (*p* = 0.452, 0.641, 0.467) (Table 1). Sixty-one patients (32.5%) in the hemithyroidectomy with RFA group and 76 patients (35.1%) in the total thyroidectomy group harbored central lymph node metastases that were more than 2 mm (*p* = 0.110). In the hemithyroidectomy with the RFA group, no patient received radioactive iodine. Thirteen (6.8%, 13/191) patients who underwent total thyroidectomy were confirmed to have more than 5 metastatic lymph nodes, 11 of whom received radioactive iodine. Compared to contralateral lobectomy during total thyroidectomy, which took 20–30 min, hemithyroidectomy with RFA took 5–10 min to perform. Nine of the 191 patients who underwent total thyroidectomy were diagnosed with contralateral PTC (seven micro-PTCs, two 20 mm).

**Table 1:** Baseline characteristics of participants

Characteristics	Before matching			After matching		
	Hemithyroidectomy with RFA	Total thyroidectomy	*P* value	Hemithyroidectomy with RFA	Total thyroidectomy	*P* value
Cases	271	368		191	191	
Age at surgery	41 (21–54)	42 (25–50)	0.043	32 (24–50)	34 (21–51)	0.364
Female	188 (69.4%)	265 (72.0%)	0.468	103 (53.9%)	98 (51.3%)	0.608
Primary tumor						
Right lobe	154 (56.8%)	222 (40.8%)	0.374	116 (60.7%)	109 (57.1%)	0.467
Multifocality	29 (10.7%)	41 (11.1%)	0.860	19 (9.9%)	21 (11.0%)	0.738
Nodule diameter (cm)	1.4 (1.0 – 3.4)	1.8 (1.0 – 4.0)	0.012	1.6 (1.0 – 3.5)	1.7 (1.1 – 4.0)	0.751
Contralateral nodule(s)						
Multifocality	84 (31.0%)	150 (40.7%)	0.011	48 (25.1%)	51 (26.7%)	0.656
Nodule diameter	0.8 ± 0.7	1.0 ± 0.9	0.017	0.9 ± 0.8	1.0 ± 0.7	0.452
≥ 1.0 cm	57 (21.0%)	114 (31.0%)		57 (21.0%)	114 (31.0%)	0.726
Nodule volume (ml)	3.0 ± 2.9	3.4 ± 2.4	0.020	3.1 ± 2.6	3.3 ± 2.1	0.641
Pathological lymph node metastasis	80 (29.5%)	143 (38.9%)	0.014	62 (32.5%)	67 (35.1%)	0.589
Serum thyroglobulin (normal: 1.59 – 50.03) (μg/ml) (one month after surgery)	21.77 (15.03 – 37.79)	0.07 (0.05–2.23)	0.247	22.81 (16.27 – 34.21)	0.41 (0..07 – 1.85)	0.127
Median follow-up period	4.1 years (2.0–5.4)	4.7 years (2.0–6.1)	0.024	4.1 years (2.0–5.4)	4.2 years (2.0–5.5)	0.172

### Changes in benign thyroid nodules in the study group

**Fig. 2:**
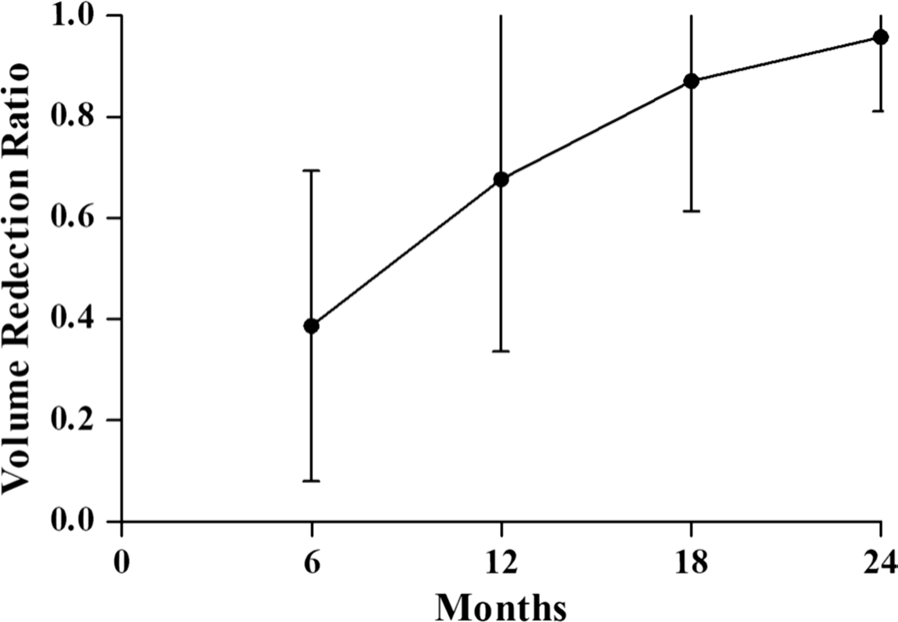
Volume Reduction Ratio at each follow up

Forty-eight (25.1%) patients in the hemithyroidectomy with RFA group had multiple nodules in the contralateral lobe. An average of 2 (1–4) nodules were ablated in the contralateral lobe. All the nodules on the contralateral lobe were benign. The FNAC outcome for 25 contralateral nodules (25/224, 11.2%) was indeterminate, and FNAC was performed twice. The initial mean size of the contralateral nodule was 0.9 ± 0.8 cm (Table 1). The average volume of 224 nodules was 3.1 ± 2.6 ml before treatment, 1.9 ± 1.8 ml at 6 months, 1.0 ± 0.6 ml at 12 months, and 0.4 ± 0.2 at 18 months and 0.13 ± 0.1 at the final follow-up period. A second ablation was performed on thirty-three (14.7%) nodules. The nodular volume decreased progressively, and the differences were statistically significant compared with the volume before treatment (*p* < 0.001). The VRR gradually increased by 38.7% at 6 months, 67.7% at 12 months, 87.1% at 18 months and 95.8% at the final follow-up period (Fig. 2).

### Complications

In the hemithyroidectomy with intraoperative RFA group, transient recurrent laryngeal nerve injury was confirmed in one patient (0.5%) on the side of the hemithyroidectomy. In contrast, four (2.1%) were documented in the total thyroidectomy group (*p* = 0.386). These patients recovered within six months. The injured nerves recovered, and the vocal cords moved fully in adduction and abduction. In the total thyroidectomy group, one (1/191, 0.5%) patient had permanent recurrent laryngeal nerve injury, with persistent dysphonia and documented palsy more than 6 months after surgery. In the hemithyroidectomy with RFA group, five (2.6%) had transient hypoparathyroidism. None of the patients had permanent hypoparathyroidism with low serum calcium and PTH after six months of continuous use of calcium tablets and vitamin D. Conversely, of the patients who underwent total thyroidectomy, 15 (7.8%) and three (1.6%) developed transient and permanent hypocalcemia with the use of medicine within one year and beyond one year, respectively (Table 2).

**Table 2:** Adverse events between hemithyroidectomy plus intraoperative RFA and total thyroidectomy during the follow up

	Hemithyroidectomy with RFA (*n* = 191)	Total thyroidectomy (*n* = 191)	*P* value
Hypocalcemia (within six months)	5 (2.6)	15 (7.8)	0.022
Hypocalcemia (beyond six months)	0	3 (1.6)	0.246
Recurrent laryngeal nerve paralysis (within six months)	1 (0.5)	4 (2.1)	0.368
Recurrent laryngeal nerve paralysis (beyond six months)	0	1 (0.5)	1.000
locoregional recurrence	3 (1.6)	0	0.246
New lesions	2 (1.1)	0	0.478
Lymph node metastasis	1	0	1.000

*RFA* Radiofrequency ablation

### Postoperative supplemental levothyroxine therapy

TSH was maintained in the mid to lower reference range (0.5–2 mU/L), while surveillance for recurrence was continued. Thyroid hormone therapy was unnecessary if patients could maintain their serum TSH in this target range. The rate of patients who were not required to receive supplemental levothyroxine while under surveillance after hemithyroidectomy with intraoperative RFA was 28.3% (54/191) within one year. The normal range of TSH in our institution was 0.3–4.6 mU/L. A total of 103 (53.9%, 103/191) patients received levothyroxine for TSH suppression with TSH levels ≤ 4.6 mU/L, and 34 (17.8%, 34/191) patients received levothyroxine for hypothyroidism with TSH levels > 4.6 mU/L one year after surgery.

### Disease recurrence

In the hemithyroidectomy and RFA groups, two patients (2.1%) had new lesions (1.2 cm and 1.4 cm) that were cancerous, arising in the remaining thyroid of the contralateral lobe. The time to recurrence was 4.1 years and 3.5 years for the two patients with 2.4 cm and 2.9 cm primary PTCs who encountered recurrences on the contralateral lobe. One case (1/191, 0.5%) harbored cervical lymph node metastasis (9 mm) in the untreated contralateral central neck 3.0 years after surgery, and no recurrence was observed in the remnant thyroid lobe. All three patients underwent completion thyroidectomy and central neck dissection on the contralateral lobe. No scar tissue or adhesion between the strap muscles and the thyroid gland was observed following completion thyroidectomy. With the help of intraoperative neuromonitoring, no RLN injury occurred in any of the three cases. None of the patients encountered hypocalcemia after a completion thyroidectomy.

The median levels of serum thyroglobulin (Tg) were 22.81 μg/ml (16.27–34.21) in the hemithyroidectomy with RFA group and 0.41 μg/ml (0.07–1.85) in the total thyroidectomy group one month after surgery. Tg levels in the three patients undergoing hemithyroidectomy with RFA who encountered locoregional recurrence were not obviously increased.

## Discussion

For unilateral PTC with benign thyroid nodules on the opposite side, the optimal extent of surgery remains controversial [21]. This propensity score matching study compared hemithyroidectomy plus intraoperative RFA with total thyroidectomy concerning complications and morbidity after treatment. The results of this study indicated that hemithyroidectomy plus intraoperative RFA was comparable to total thyroidectomy with respect to oncologic efficacy after a median follow-up of 4.1 years. Intraoperative RFA effectively decreased the volume of contralateral nodules (95.8% volume reduction at the 2-year follow-up). The rate of transient hypocalcemia was significantly lower after hemithyroidectomy with RFA than following total thyroidectomy.

According to the 2015 ATA guidelines, patients receive thyroid lobectomy for thyroid cancer and observation with regular follow-up for contralateral benign nodules. Some patients were anxious about contralateral progression to malignancy after thorough patient education. These patients tend to choose subtotal or total thyroidectomy. It provides a definitive cure of the disease and the promise of total relief of any compressive symptoms associated with it. Although total thyroidectomy is widely available and generally safe, there are still risks of complications, such as the high frequency of hypocalcemia requiring vitamin D treatment. Hemithyroidectomy benefits included avoiding the risk of temporary or permanent hypoparathyroidism and potentially halving the risk of superior and recurrent laryngeal nerve injury [22]. Under these circumstances, hemithyroidectomy for the lobe with malignant thyroid nodules and intraoperative RFA for the contralateral lobe with benign thyroid nodules could be a compromise.

In the hemithyroidectomy with intraoperative RFA group, we found that the volume of all thyroid nodules significantly decreased after treatment (Fig. 2). Furthermore, no serious complications were observed after treatment. Considering that patients in China tend to have anxiety of nodules being malignant and the potential to undergo a second operation, intraoperative RFA was an alternative method to ablate the contralateral benign thyroid nodule. Thus, hemithyroidectomy with intraoperative RFA was proposed to decrease the incidence of hypocalcemia and RLN injury, relieving the anxiety of disease progression.

In the present study, the findings revealed that the total thyroidectomy group had a higher rate of transient hypocalcemia than the total thyroidectomy group (7.8% vs. 2.6%, *p* = 0.022). Transient hypocalcemia occurred in five (2.6%) patients undergoing hemithyroidectomy with RFA. This might have resulted from RFA damage to the parathyroid gland, and the calcium value recovered within three months. The hemithyroidectomy with intraoperative RFA group contained three patients who underwent completion thyroidectomy because of the recurrence of the contralateral primary nodule. However, the risk of thyroid cancer recurrence and the need for a second surgery impacted patients’ preference for treatment options. For unilateral PTC patients with a normal contralateral lobe treated with lobectomy, the rate of complete thyroidectomies was 9.7%. The rate of contralateral cancer was 4.1%, with a mean follow-up of 6.8 years [23]. Compared with the 10% completion thyroidectomy followed by lobectomy, 2.1% of patients in the hemithyroidectomy with intraoperative RFA group underwent completion thyroidectomy.

Hypothyroidism requiring thyroid hormone replacement occurs in 10% to 48% of patients after lobectomy [24]. Suppose exogenous hormone supplementation was not adequate for any reason. In that case, the residual thyroid lobe could still produce some thyroid hormone, unlike patients after total thyroidectomy, who were completely dependent on exogenous hormone intake as the only source of thyroid hormone. In the present study, 54 patients (28.3%) in the hemithyroidectomy with intraoperative RFA group did not require supplemental levothyroxine while under surveillance.

There were several limitations in the propensity score matching study. First, it was a prospective study and retrospective review. To eliminate the bias of age, sex, and tumor volume, we utilized propensity score matching to compare the two groups. However, there still exists a significant risk for selection bias. The group that underwent total thyroidectomy could have had higher clinical suspicion of contralateral disease. Conversely, the group undergoing hemithyroidectomy with RFA could have had lower clinical suspicion of disease. Second, the median follow-up was 4.1 years, and the incidence of recurrence might be underestimated. The recurrence between the two groups might have no significant difference with a longer follow-up in reference to the comparison between lobectomy and total thyroidectomy. Appropriate patient education, including thorough decision aids regarding the clinical significance or insignificance of a given finding, would be a better pathway than reducing the patient's anxiety.

## Conclusions

In conclusion, while unilateral PTC patients with contralateral benign nodules were anxious about a malignant change and wished to get rid of the lesion, intraoperative RFA could be applied to treat the contralateral nodule according to the patients' wishes. This treatment strategy helps patients relieve anxiety and decrease the risks of complications from total thyroidectomy.

## Data Availability

Not applicable.

## Abbreviations

PTC: Papillary thyroid carcinoma
RFA: Radiofrequency ablation
HTRFA: Hemithyroidectomy with intraoperative RFA
HADS: Hospital anxiety and depression scale
FNAC: Fine-needle aspiration cytology
TSH: Thyroid stimulating hormone
VCP: Vocal cord paralysis
